# Retrospective review of risk factors for surgical wound dehiscence and incisional hernia

**DOI:** 10.1186/s12893-017-0207-0

**Published:** 2017-02-22

**Authors:** Sofie Walming, Eva Angenete, Mattias Block, David Bock, Bodil Gessler, Eva Haglind

**Affiliations:** 1 Department of Surgery, Institute of Clinical Sciences, Sahlgrenska Academy, University of Gothenburg, SSORG – Scandinavian Surgical Outcomes Research Group, Sahlgrenska University Hospital/Östra, 416 85 Gothenburg, Sweden; 2 Department of Surgery, Institute of Clinical Sciences, Sahlgrenska Academy, University of Gothenburg, Sahlgrenska University Hospital/Östra, 416 85 Gothenburg, Sweden

**Keywords:** Hernia, Laparotomy, Risk factors, Surgical wound dehiscence

## Abstract

**Background:**

Several factors and patient characteristics influence the risk of surgical wound dehiscence and incisional hernia after midline laparotomy. The purpose of this study was to investigate whether a specified, or not specified, suture quota in the operative report affects the incidence of surgical wound complications and to describe the previously known risk factors for these complications.

**Methods:**

Retrospective data collection from medical records of all vascular procedures and laparotomies engaging the small intestines, colon and rectum performed in 2010. Patients were enrolled from four hospitals in the region Västra Götaland, Sweden. Unadjusted and adjusted Cox regression analyses were used when calculating the impact of the risk factors for surgical wound dehiscence and incisional hernia.

**Results:**

A total of 1,621 patients were included in the study. Wound infection was a risk factor for both wound dehiscence and incisional hernia. BMI 25–30, 30–35 and >35 were risk factors for wound dehiscence and BMI 30–35 was a risk factor for incisional hernia. We did not find that documentation of the details of suture technique, regarding wound and suture length, influenced the rate of wound dehiscence or incisional hernia.

**Conclusions:**

These results support previous findings identifying wound infection and high BMI as risk factors for both wound dehiscence and incisional hernia. Our study indicates the importance of preventive measures against wound infection and a preoperative dietary regiment could be considered as a routine worth testing for patients with high BMI planned for abdominal surgical precedures.

## Background

A midline incision is often used in colorectal and vascular procedures. By using this approach ample access to the abdominal cavity is achieved with limited damage to the muscles, nerves, and blood supply of the abdominal wall. Wound complications such as surgical wound infection, wound dehiscence and incisional hernia are common, cause patient suffering and generate prolonged hospital stay [1, 2]. Experimental and clinical evidence indicate that wound dehiscence and incisional hernia are related to the surgical technique used at wound closure [3–5]. Accordingly, the surgeon can to some extent control the risk for wound complications.

Jenkins was the first to propose a ratio of 4:1 between the length of the suture and the length of the surgical wound [6]. Later, Israelsson et al. confirmed Jenkin’s hypothesis and also proposed that the length of the suture and the length of the surgical wound should be measured and noted in the surgical notes at each abdominal wall closure [7, 8].

Additional risk factors for wound dehiscence and the development of an incisional hernia directly related to the patient have previously been described and include male gender [1], local wound infection [5, 9], obesity [10], the use of glucocorticosteroids [2, 11], hypoalbuminemia, anemia and emergency operations [12].

The primary objective of this study was to compare the rate of wound dehiscence and incisional hernia formation following Jenkins’ 4:1 closure technique as it was documented in the operational report. Since the ratio is not always stated in the notes from the operation, the hypothesis was that if the ratio between the suture length and the wound length is stated, the surgeon has focused on the closure technique, thus affecting the risk for wound complications.

The aim was to investigate if there was an association between the documentation in the medical record of a suture length to wound length ratio, and the incidence of wound complications. An additional objective was to assess the significance of the previously described risk factors for surgical wound complications.

## Methods

**Fig. 1:**
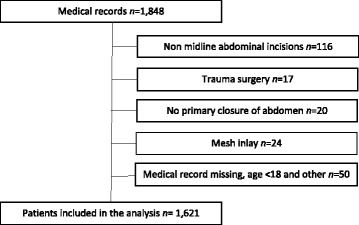
Flow chart

The data in this study were gathered from 4 hospitals serving 1,600,000 inhabitants in western Sweden: Sahlgrenska University Hospital, Göteborg; NU Hospital Group, Trollhättan; Skaraborg Hospital, Skövde and Södra Älvsborg Hospital, Borås. All patients who underwent primary or secondary laparotomy through midline abdominal incisions for vascular procedures or laparotomies with drainage or lavage, procedures on the small bowel, the colon or the rectum between January 1, 2010 and December 31, 2010 were included. The patients were identified using codes from the Nordic Medico-Statistical Committee (NOMESCO) Classification of Surgical Procedures version 1.9. Exclusion criteria were trauma surgery, no initial closure of the abdominal wall and patients with primary mesh inlay at the midline abdominal incision. To conform with the hypothesis we excluded the patients where a documented suture quota <3.5 was stated in the operative report (*n =* 4), since such a low ratio cannot be considered clinically acceptable (Fig. 1).

A clinical record form (CRF) was constructed and used for data extraction from medical records regarding suture technique (specified ratio ≥3.5:1 or unspecified ratio, hereafter referred to as *specified group* and *unspecified group*), emergency or elective procedure, demography, co-morbidity, type of surgery, suture technique, surgical wound complications, reoperations and mortality. Skin preparation was by washing with antibacterial agents 1–3 times before surgery and local skin disinfection in the operation theatre was performed according to standard practice. Factors that were not possible to ascertain retrospectively were suture type, how many patients were given antibiotics pre- or post-operation, length of surgery or the surgeon’s experience level. Wound complications of interest were wound infection, wound dehiscence and incisional hernia. Data were extracted in 2014, by one of the authors (SW).

Each patient was followed from the index operation in 2010 until the time of the review of the medical records in 2014, rendering a follow-up time with a median of forty-one months (range 0–58). The end of the follow-up was defined by one of the following: the time of review of the medical record, a renewed operation with midline abdominal incision, death of the patient or if the patient was lost to follow-up.

### Definition of endpoints

Wound dehiscence was defined as a complete disruption of the wound including the fascia closure after the index operation or by a significant gap between the edges of the fascia necessitating reoperation. Incisional hernia was defined as documentation of hernia in the medical records or re-operation for this condition. Registration was based on clinical findings and did not depend on a CAT scan having been done. Timing of the occurrence of wound dehiscence and incisional hernia was retrieved from the medical records. The definition used for wound infection was based on two factors: records noting that the patient was treated with antibiotics for wound infection or if there was a note in the medical record of purulent discharge from the wound, irrespective of positive bacteriologic cultures or treatment with antibiotics.

### Statistical methods

To assess the relationship between suture technique and occurrence of reported wound dehiscence and incisional hernia, as well as the significance of the previously documented risk factors in the studied cohort, a Cox proportional hazards model [13] was used after checking the validity of model assumptions [14]. Risk factors found to have an eligible contribution, defined as having a *p*-value for the Wald test <.20, were simultaneously included in a multiple Cox regression analysis. As our primary objective was to evaluate the significance of suture technique, this risk factor was included in all analyses. Statistical analyses were performed using SAS 9.3 (SAS Institute Incorporated, Cary, NC, USA) and R (R Development Core Team. A language and environment for statistical computing. Vienna, Austria: R Foundation for Statistical Computing; 2005).

## Results

We identified 1,848 patients and after applying the exclusion criteria 1,621 patients remained (Fig. 1). Patient characteristics are shown in Table 1. The patients in the unspecified group (suture quota not documented) were more often operated on as an emergency procedure and more often had a Body Mass Index (BMI) >25. There was a higher frequency of reported smokers in the unspecified group, however, data on smoking were relatively often missing (*n = 255*) in the medical records. Overall, 147 (9.0%) in the patient cohort had surgical wound infection, 59 (10.2%) in the specified group (suture quota ≥3.5 documented) and 88 (8.4%) in the unspecified group. The patients include 748 patients operated on for malignant diseases, 98 with vascular diseases and 773 with other diseases such as ileus, Crohn’s disease and ulcerative colitis. Overall mortality at follow-up were 522 (32.1%) of 1,621 patients.

**Table 1:** Demographics for patients (*n* = 1,621)

	Suture technique, as stated in medical record		
	Suture quota ≥3,5 stated *Specified group*	Suture quota not stated *Unspecified group*	Total
	*n=*	*n=*	*n=*
Number of patients	592	1,029	1,621
Age at index operation^a^	67.8 (17.8–94.4)	71.0 (18.1–97.5)	70.1 (17.8–97.5)
Sex (M : F)	285 : 307	516 : 513	801 : 820
Emergency operation (*n* = 1601)^c^	187 (31.6)	510 (49.5)	697 (43.5)
Surgical wound infection	59 (10.0)	88 (8.6)	147 (9.1)
Smoking (*n* = 1,370)^c^	114 (20.5)	205 (25.2)	319 (23.3)
BMI > 25 (*n =* 1,361)^c^	244 (44.8)	401 (49.3)	645 (47.4)
Malignant disease	297 (50.2)	451 (43.8)	748 (46.1)
Comorbidity			
Diabetes mellitus	79 (13.3)	153 (14.9)	232 (14.3)
Cardiovascular disease^b^	89 (15.0)	206 (20.0)	295 (18.2)
Renal failure	10 (1.7)	39 (3.8)	49 (3.0)
COPD	39 (6.6)	81 (7.9)	120 (7.4)
Steroid usage	44 (7.4)	69 (6.7)	113 (7.0)

Values in parenthesis are percentages unless indicated

^a^Years in median (range)

^b^Myokardial infarction, heart failure, angina pectoris or intermittent claudication

^c^All data were not available for all patients, evaluable number of patients is stated in each row

Ninety-eight patients underwent vascular surgery: 89 for abdominal aortic aneurysm, 7 for aortoiliac occlusive disease and 2 for iliac artery aneurysm. Wound dehiscence affected 4 of these patients, all operated on for abdominal aortic aneurysm. Nine patients developed incisional hernia, 7 of which were operated on for abdominal aortic aneurysm.

Sixty-one patients developed wound dehiscence, 19 (3.3%) in the specified group and 42 (4.0%) in the unspecified group. Fifty-three (86.9%) patients who had surgical wound dehiscence were reoperated for their wound dehiscence. Eight (13.1%) patients with documented wound dehiscence later developed incisional hernia. Twenty-eight (45.9%) patients with wound dehiscence were deceased at follow-up.

Incisional hernia developed in 105 patients, 33 (5.6%) in the specified group and in 76 (7.4%) in the unspecified group. According to the medical records 46 (43.8%) patients with incisional hernia were surgically treated. With regard to the primary objective, to investigate if there was an association between documentation of suture length to wound length ratio and the incidence of wound complications, no statistical significance was seen in the unadjusted analysis regarding either wound dehiscence or incisional hernia (Table 2 *)*.

**Table 2:** Analysis of risk factors for wound dehiscence and incisional hernia with unadjusted Cox Regression

	End point				
Risk factor	Wound Dehiscence		Incisional Hernia		
	HR 95% CI	*p* ^a^	HR 95% CI	*p* ^a^	*n=*
Suture technique, specified vs. not specified	1.31 (0.76–2.26)	0.324	1.44 (0.95–2.18)	0.086	1,621
Wound infection	3.00 (1.65–5.46)	<0.001	3.68 (2.38–5.71)	<0.001	1,621
Sex, male vs. female	1.98 (1.17–3.36)	0.011	1.14 (0.78–1.67)	0.516	1,621
Priority of operation, emergency vs. scheduled	1.62 (0.98–2.68)	0.060	1.22 (0.82–1.81)	0.327	1,601
Smoking	1.61 (0.93–2.79)	0.091	1.15 (0.77–1.71)	0.506	1,370
BMI 25–30 vs <25	1.00 (0.50–2.00)	0.029	2.19 (1.34–3.58)	<0.001	1,356
BMI 30–35 vs <25	2.62 (1.29–5.32)		2.63 (1.43–4.83)		
BMI >35 vs <25	2.17 (0.65–7.29)		4.81 (1.19–10.60)		
Hypoalbuminemia (S-alb <35)	0.95 (0.51–1.75)	0.867	0.77 (0.47–1.24)	0.275	757
Anemia (S-Hb <100)	0.62 (0.35–1.11)	0.110	0.70 (0.43–1.12)	0.138	1,480
Diabetes	1.36 (0.71–2.60)	0.356	1.08 (0.63–1.87)	0.779	1,621
Cardiovascular disease*	2.03 (1.17–3.52)	0.012	1.46 (0.92–2.31)	0.112	1,621
Renal failure	2.49 (0.90–6.87)	0.077	0.48 (0.07–3.41)	0.459	1,621
COPD	2.66 (1.35–5.23)	0.005	1.16 (0.54–2.50)	0.701	1,621
Peroral cortison at intake	1.53 (0.66–3.55)	0.323	1.07 (0.47–2.45)	0.868	1,621

^a^Wald test of regressions coefficient

*﻿Myokardial infarction, heart failure, angina pectoris or intermittent claudication

Risk factors in the unadjusted analysis for wound dehiscence were wound infection, male gender, BMI 30–35, cardiovascular disease and chronic obstructive pulmonary disease (COPD). The risk factors for incisional hernia were wound infection and BMI 25–30, BMI 30–35 and BMI >35 (Table 2).

In the adjusted analysis wound infection was identified as a risk factor for both wound dehiscence (*p* = 0.020) and incisional hernia (*p* = <0.001). For incisional hernia BMI 30–35 was a risk factor (*p* = 0.002). For wound dehiscence the risk factors were BMI 25–30 (*p* = 0.001), BMI 30–35 and BMI >35 (Table 3). There were no significant differences regarding the specified and unspecified group for any of the end-points (Table 3).

**Table 3:** Analysis of risk factors for wound dehiscence and incisional hernia with adjusted Cox Regression

Risk factor	End point	
	Wound Dehiscence	
	HR 95% CI	*p* ^b^
Suture technique, specified vs. not specified	1.36 (0.73–2.53)	0.340
Wound infection	2.33 (1.14–4.77)	0.020
Sex, male vs. female	1.69 (0.92–3.12)	0.092
Priority of operation, emergency vs. scheduled	1.51 (0.81–2.81)	0.192
Smoking	1.32 (0.74–2.34)	0.304
BMI 25–30 vs <25	0.82 (0.39–1.73)	0.025
BMI 30–35 vc <25	2.57 (1.23–5.36)	
BMI >35 vs <25	1.85 (0.54–6.34)	
Anemia, S-Hb < 100 vs. S-Hb > 100	0.63 (0.31–1.28)	0.203
Cardiovascular disease^a^	1.48 (0.74–2.97)	0.271
Renal failure	0.50 (0.07–3.79)	0.501
COPD	1.29 (0.49–3.42)	0.605
	Incisional Hernia	
	HR 95% CI	*p* ^b^
Suture technique, specified vs. not specified	1.37 (0.88–2.13)	0.166
Wound infection	3.47 (2.16–5.56)	<0.001
BMI 25–30 vs <25	2.11 (1.29–3.45)	0.001
BMI 30–35 vc <25	2.41 (1.31–4.43)	
BMI >35 vs <25	3.87 (1.74–8.61)	
Anemia, S-Hb < 100 vs. S-Hb > 100	0.85 (0.48–1.48)	0.559
Cardiovascular disease^a^	1.35 (0.81–2.26)	0.247

^a^Myokardial infarction, heart failure, angina pectoris or intermittent claudication. ^b^ Wald test of regressions coefficient

## Discussion

The analysis of the possible risk factors for surgical wound dehiscence and incisional hernia support previous findings identifying BMI 30–35 as a risk factor for wound dehiscence and BMI ≥25 as risk factor for wound dehiscence and incisional hernia [2, 15, 16]. It is possible that the risk of incisional hernia increases with high BMI. However, information on BMI was often missing and the results should be interpreted with caution. We found that wound infection was a risk factor for both endpoints. This has been suggested in several reports previously [1, 2, 5], however, conflicting results have also been presented [9]. Niggebrugge et al. [11] could not find such a relationship. They did, however find that prophylactic antibiotics reduced the risk for wound dehiscence. Our study also indicated the importance of preventive measures against wound infection.

This study adds new information about the incidence of wound dehiscence. This wound complication has not previously been identified by review of medical records from both elective and emergency surgery, which may explain why the incidence of wound dehiscence was higher than the literature gave reason to expect [1, 2, 7, 17].

According to previously published studies the technique used at closure of midline abdominal incisions affected the rates of incisional hernia [3, 4, 7]. In 2010, the routine to calculate and document the suture quota had not yet been fully adopted at all 4 hospitals that participated in this study. However, we did not find that documentation of the details of suture technique, regarding wound and suture length, influenced the rate of wound dehiscence or incisional hernia. Since we do not know which technique was actually used when documentation of suture quota was lacking in the medical records, the results of this study do not contradict the results of previous studies.

High BMI has previously been reported to be associated with a significant increase in complication rates within 30 days after colorectal cancer surgery [10]. In bariatric surgery a preoperative dietary regimen is routinely used to ensure weight loss in order to decrease perioperative complications [18]. Whether this routine also decreased wound dehiscence and incisional hernia is still unclear.

The strengths of our study lie in the population basis and the large cohort, the fact that the cohort was consecutive including both elective and emergency operations, the short inclusion time and the long follow-up period. It has previously been found that it is important to monitor incisional hernias at least 3 years after surgery, as short-term follow-up could underestimate the incidence [19]. The endpoints were defined before retrieval of data and we used a specific clinical record form (CRF).

The study design has certain limitations, the most important being the retrospective data retrieval from medical records. Using a small bites suture technique rather that a large bites technique has previously been reported to affect the incidence of incisional hernia [17]. Our study could not consider aspects of the suture technique other than the suture quota since we were restricted to the information given in the medical records. Another limitation was that the patients were not specifically examined for the occurrence of an incisional hernia during the follow up, and the only incisional hernias recorded were those noted in the medical records. The incidence we found may thus be lower than the actual incidence. However, the rate corresponded to previous reports of clinically relevant incisional hernias [4, 5, 7].

## Conclusion

In conclusion we cannot demonstrate that surgical technique, as described in surgical notes, had an impact on wound dehiscence and later incisional hernia. Other risk factors for these complications, according to our analyses, were wound infection and high BMI. Therefore we suggest that all evidence-based precautions should be taken to avoid wound infections. A preoperative dietary regimen could be considered as a routine worth testing for patients with high BMI planned for other abdominal surgical procedures than bariatric surgery.

## Abbreviations

BMI: Body Mass Index
COPD: Chronic obstructive pulmonary disease
CRF: Clinical record form

## References

[1] Gislason H, Gronbech JE, Soreide O (1995). Burst abdomen and incisional hernia after major gastrointestinal operations--comparison of three closure techniques. Eur J Surg.

[2] Riou JPA, Cohen JR, Johnson H (1992). Factors influencing wound dehiscence. Am J Surg.

[3] Gruppo M, Mazzalai F, Lorenzetti R, Piatto G, Toniato A, Ballotta E (2012). Midline abdominal wall incisional hernia after aortic reconstructive surgery: a prospective study. Surgery.

[4] Varshney S, Manek P, Johnson CD (1999). Six-fold suture:wound length ratio for abdominal closure. Ann R Coll Surg Engl.

[5] Gislason H, Soreide O, Viste A (1999). Wound complications after major gastrointestinal operations. The surgeon as a risk factor. Dig Surg.

[6] Jenkins TP (1976). The burst abdominal wound: a mechanical approach. Br J Surg.

[7] Israelsson LA, Jonsson T (1993). Suture length to wound length ratio and healing of midline laparotomy incisions. Br J Surg.

[8] Israelsson LA (1999). Incisional hernias in patients with aortic aneurysmal disease: The importance of suture technique. Eur J Vasc Endovasc Surg.

[9] Diener MK, Knebel P, Kieser M, Schuler P, Schiergens TS, Atanassov V (2014). Effectiveness of triclosan-coated PDS Plus versus uncoated PDS II sutures for prevention of surgical site infection after abdominal wall closure: the randomised controlled PROUD trial. Lancet.

[10] Hede P, Sorensson MA, Polleryd P, Persson K, Hallgren T (2015). Influence of BMI on short-term surgical outcome after colorectal cancer surgery: a study based on the Swedish national quality registry. Int J Colorectal Dis.

[11] Niggebrugge AH, Trimbos JB, Hermans J, Steup WH, Van De Velde CJ (1999). Influence of abdominal-wound closure technique on complications after surgery: a randomised study. Lancet.

[12] Makela JT, Kiviniemi H, Juvonen T, Laitinen S (1995). Factors influencing wound dehiscence after midline laparotomy. Am J Surg.

[13] Cox DR (1972). Regression models and life-tables. J R Stat Soc.

[14] Grambsch PMT (1994). T M Proportional hazards tests and diagnostics based on weighted residuals. Biometrika.

[15] Israelsson LA, Jonsson T (1997). Overweight and healing of midline incisions: the importance of suture technique. Eur J Surg.

[16] Henriksen NA, Helgstrand F, Vogt KC, Jorgensen LN, Bisgaard T (2013). Risk factors for incisional hernia repair after aortic reconstructive surgery in a nationwide study. J Vasc Surg.

[17] Deerenberg EB, Harlaar JJ, Steyerberg EW, Lont HE, van Doorn HC, Heisterkamp J (2015). Small bites versus large bites for closure of abdominal midline incisions (STITCH): a double-blind, multicentre, randomised controlled trial. Lancet.

[18] Edholm D, Kullberg J, Karlsson FA, Haenni A, Ahlstrom H, Sundbom M (2015). Changes in liver volume and body composition during 4 weeks of low calorie diet before laparoscopic gastric bypass. Surg Obes Relat Dis.

[19] Fink C, Baumann P, Wente MN, Knebel P, Bruckner T, Ulrich A (2014). Incisional hernia rate 3 years after midline laparotomy. Br J Surg.

